# Characterization of Estrogen and Androgen Activity of Food Contact Materials by Different *In Vitro* Bioassays (YES, YAS, ERα and AR CALUX) and Chromatographic Analysis (GC-MS, HPLC-MS)

**DOI:** 10.1371/journal.pone.0100952

**Published:** 2014-07-07

**Authors:** Johannes Mertl, Christian Kirchnawy, Veronica Osorio, Angelika Grininger, Alexander Richter, Johannes Bergmair, Michael Pyerin, Michael Washüttl, Manfred Tacker

**Affiliations:** OFI - Austrian Research Institute for Chemistry and Technology, Vienna, Austria; Leiden University Medical Centre, Netherlands

## Abstract

Endocrine active substances (EAS) show structural similarities to natural hormones and are suspected to affect the human endocrine system by inducing hormone dependent effects. Recent studies with *in vitro* tests suggest that EAS can leach from packaging into food and may therefore pose a risk to human health. Sample migrates from food contact materials were tested for estrogen and androgen agonists and antagonists with different commonly used *in vitro* tests. Additionally, chemical trace analysis by GC-MS and HPLC-MS was used to identify potential hormone active substances in sample migrates. A GC-MS method to screen migrates for 29 known or potential endocrine active substances was established and validated. Samples were migrated according to EC 10/2011, concentrated by solid phase extraction and tested with estrogen and androgen responsive reporter gene assays based on yeast cells (YES and YAS) or human osteoblast cells (ERα and AR CALUX). A high level of agreement between the different bioassays could be observed by screening for estrogen agonists. Four out of 18 samples tested showed an estrogen activity in a similar range in both, YES and ERα CALUX. Two more samples tested positive in ERα CALUX due to the lower limits of detection in this assay. Androgen agonists could not be detected in any of the tested samples, neither with YAS nor with AR CALUX. When testing for antagonists, significant differences between yeast and human cell-based bioassays were noticed. Using YES and YAS many samples showed a strong antagonistic activity which was not observed using human cell-based CALUX assays. By GC-MS, some known or supposed EAS were identified in sample migrates that showed a biological activity in the *in vitro* tests. However, no firm conclusions about the sources of the observed hormone activity could be obtained from the chemical results.

## Introduction

Food contact materials have been identified as a possible source of endocrine active substances (EAS) that may interfere with the human endocrine system [1]. EAS can act as endocrine disrupting chemicals (EDCs), that may alter the activity of natural steroid hormones by modifying their regulatory pathways, interacting with steroid receptors and antagonizing endogenous hormones, or simply mimicking steroid hormone dependent effects [2]. Physiological processes such as reproduction, cell growth and regulation of glucose levels are generally regulated by steroid hormones and are therefore vulnerable to EDC effects [3]. Many *in vivo* studies with mammals have linked an increased EDC intake to a variety of reproductive and physiological abnormalities such as reduced sperm quality in males, feminization of laboratory animals, obesity, or the development of breast, ovarian and prostate cancers [4]–[7]. These studies have increased awareness and concern about possible health impairment in humans due to EDC-exposure, since humans have an endocrine system similar to other mammals [8]. In the past, several *in vivo* bioassays [9]–[11] and high-throughput *in vitro* bioassays have been developed [12]. Most of the *in vitro* assays are based on the activation of steroid hormone dependent receptors by EAS, followed by the activation and transcription of a reporter gene (e.g. firefly luciferase) [3], [13].

Migration of EAS from food contact materials has become widely investigated [14], [15]. Pinto and Reali [16] and Wagner and Oehlmann [17], [18] detected estrogen activity in mineral water packed into PET bottles using the yeast estrogen screen (YES) and the human cell-based proliferation assay E-Screen [19]. However, the source of contamination was not identified. It is not clear whether the source water, plastic components and detergents that contaminated the water during the production process, or the PET resin was the source of the detected endocrine activity. Böhmler et al. [20] detected estrogen activity in seven out of ten bottled water samples using the E-Screen, whereby the water source was already contaminated. Significant estrogen activity was also detected in 15 out of 31 bottled mineral water samples by the Swiss Federal Office of Public Health using an ERα (estrogen receptor alpha) CALUX (**c**hemically **a**ctivated **lu**ciferase e**x**pression) bioassay. However, no correlation between detected activity and packaging material was observed [21]. Plastic food packaging of different resin types tested positive for estrogen activity by Yang et al. [22] using the E-Screen. Yang et al. [22] assumed that possible migration processes of estrogen active monomers, plastic additives or degradation products caused the detected activity. In a previous study we analyzed plastic packaging of different resin types by combining an *in vitro* bioassay (YES) with chemical trace analysis by GC-MS and HPLC-MS to screen for estrogen agonists in food contact materials. Seven out of 41 samples tested estrogen positive in the YES. However, a direct correlation of estrogen activity in the YES, and substances identified by GC-MS and HPLC-MS was not possible [1]. Furthermore, inhibition of response to estrogen was observed in some migrates. These results are consistent with antiestrogenic effects that were detected by Wagner and Oehlmann [23] in 44% of all tested plastic packaging using the YES. Even a higher percentage of samples showed antagonistic effects in a recent study by Wagner et al. [24] analyzing different bottled water products. Antiestrogen activity was detected in 13 out of 18 water samples using the YES, whereas even 16 out of the 18 tested products showed antiandrogenic effects in the YAS [24]. It is not clear whether the observed effects were caused by antiestrogenic or antiandrogenic compounds blocking estrogen and androgen receptors, or due to unspecific inhibitions, which only occur in the yeast strain.

In the present study the observed antagonistic effects in the YES were further investigated and compared to the results of human cell-based bioassays. Phylogenetic differences in target gene activation and metabolism of steroids between yeast and mammalian cells exist [25]. Consequently, human cell-based bioassays are generally more suitable to identify substances that could influence the human endocrine system [12]. Therefore additional *in vitro* bioassays based on human cells for (anti)estrogens and (anti)androgens were established and validated.

In order to screen food contact material migrates in high-throughput analysis for (anti)estrogen and (anti)androgen active substances we used ERα CALUX and AR (androgen receptor) CALUX bioassays. The CALUX bioassays are based on a human U2-OS osteosarcoma cell line that contains a human estrogen or androgen receptor and shows a highly sensitive and selective response to natural and synthetic estrogen, or androgen active substances [26]–[28]. In the modified U2-OS cells an activated steroid hormone receptor binds to recognition sequences in the promoter region of a luciferase gene and activates luciferase transcription, which emits light when luciferin is added as a substrate. This signal increases depending on the dose of estrogen or androgen active substances [29]. In addition to screening for estrogen and androgen active substances we also analyzed antagonistic effects of food packaging migrates. Previously detected inhibiting effects of certain samples in the YES were further investigated using the ERα CALUX bioassay to determine if they were caused by estrogen antagonists [1]. Furthermore, food contact materials were also analyzed for antiandrogenic effects using a yeast androgen screen (YAS) and the AR CALUX. Both yeast bioassays (YES and YAS) have been used as standardized test methods and were used as independent test systems to compare results obtained from CALUX bioassays.

The purpose of this study was to evaluate different bioassays for their suitability to analyze food contact material migrates for their (anti)estrogen and (anti)androgen activity. Plastic food packaging of different resin types were migrated by food simulants according to EC 10/2011 with worst case scenarios being used. Migrates were concentrated by solid phase extraction and analyzed by YES, YAS, ERα CALUX and AR CALUX. Furthermore, a novel GC-MS method for 29 of the most common EAS with authorized use in food contact materials in the US and/or the EU [14] was developed and validated. Samples testing positive in at least one *in vitro* bioassay were analyzed by GC-MS and HPLC-MS in order to identify (anti)estrogen and (anti)androgen active substances.

## Materials and Methods

### Chemicals and reagents

All solvents, including ethanol (EtOH), dimethyl sulfoxide (DMSO), acetonitrile (ACN) and methanol (MetOH) were supplied by Merck. Copper (II) sulfate, Lyticase from *Athrobacter luteus* (1308 U/mg protein), chlorophenol red-β-D-galactopyranoside (CPRG), α-D-glucose monohydrate, trypsin-EDTA solution, the reference standards 17β-estradiol, 2,4-dihydroxybenzophenone, bisphenol A , nonylphenol, benzylbutylphthalate, 5α-dihydrotestosterone (DHT), 4-ortho hydroxytamoxifen (4-OHT), 2,4-di-cumylphenol, diethylhexyladipate, 2,6-bis(1,1-dimethylethyl)-4-methylphenol (BHT), dicyclohexylphthalate, diethylphthalate, 2,4-di-tert-butylphenol, dibutylphthalate (DBP), di-n-hexylphthalate (DnHP), bis (2-ethylhexyl) phtalate (DEHP), 4-chloro-3-methyl-phenol, 4-methylbenzophenone, 4,4′-thiobis(6-tert-butyl-3-methyl-phenol), 2-phenylphenol, 4-phenylphenol, 4-n-nonylphenol (NP), 2,2′-methylenebis(4-ethyl-6-tert-butylphenol), 2,2′-methylene bis(4-methyl-6-tert-butylphenol), p-cumyl phenol, triclosan, butylated hydroxyanisole (BHA), ethyl-4-hydroxy-benzoate (ethylparaben), n-propyl-p-hydroxybenzoate (propylparaben), methyl p-hydroxybenzoate (methylparaben), diethylhexyladipate, diphenyl-p-phenylenediamine, oleamide, 1,4-dichlorobenzene, 2,2-dihydroxy-4-methoxybenzophenone, 2,4-dihydroxybenzophenone, oxybenzone and all amino acids were obtained from Sigma Aldrich. 1,3-diphenylpropane was supplied by ABCR GmbH & Co. KG. Yeast minimal medium was prepared by supplementing ultrapure water with 0.17% w/v yeast nitrogen base lacking amino acids and ammonium sulfate (Difco), 2% w/v α-D-glucose monohydrate, 0.5% w/v ammonium sulfate (Roth) and the appropriate amino acids. The lacZ-master mix used to detect the β-galactosidase activity was prepared according to Wagner and Oehlmann [17]. The CALUX bioassays were performed as previously described by van der Burg et al. [27]. Cells were cultured in Dulbecco's modified Eagle's medium (Gibco) supplemented with 7.5% fetal calf serum (Gibco), 200 mg/ml G418 (Sigma Aldrich) and 0.1 U/ml of a penicillin/streptomycin solution (Lonza). The assay was performed using Dulbecco's modified Eagle's medium (without phenol red, Gibco) supplemented with 5% dextran coated charcoal stripped fetal calf serum, an 0.1 U/ml penicillin/streptomycin solution. Steady Glo Luciferase assay substrate and buffer were purchased from Promega. Oasis HLB cartridges (5 cc/200 mg) were obtained from Waters Chromatography. Ninety-six well microtiter plates for cell culture were supplied by Sarstedt.

### Samples

For the purpose of this study food contact material-grade polyethylene terephthalate (PET), polypropylene (PP), polyethylene (PE), polystyrene (PS), composite films (CF), and food cartons (FC) were analyzed for their (anti)estrogen and (anti)androgen activity. Plastic samples were either received directly from packaging manufacturers or empty packaging was provided by food retailers. Packaging included different product types (bottles, foils, trays and granulates). None of the samples had been in contact with food prior to testing and care was taken to avoid any contamination of samples during sampling, storage or transport. Selected hormone active, or cytotoxic samples analyzed by Kirchnawy et al. [1] were retested in order to compare the suitability of yeast and human cell-based bioassays for packaging analysis. Antagonistic effects of specific samples previously detected in the YES were also further investigated using the CALUX bioassays.

### Migration

Migration experiments were conducted on the basis of the Regulation of the European Commission No 10/2011 and EN 1186 as previously described by Kirchnawy et al. [1]. Briefly, different resin types of food packaging were migrated with food simulants for 10 days at 60°C. Food simulants were chosen with respect to the Regulation of the European Commission No 10/2011 depending on the food type for each packaging, which is shown in Table 1. From every sample two independent migrates were prepared, subsequently concentrated by solid phase extraction and tested in the bioassays. If one or both of these first migrates tested positive for estrogen activity, further two independent migrates were analyzed in order to confirm the initial results. For each migration a solvent blank of each food simulant was migrated in a glass bottle with a polytetrafluorethylene-coated cap. Prior to solid phase extraction, 1 ml and 20 ml of each migrate were set aside for HPLC and GC analysis.

**Table 1:** Food simulants for the migration of packaging samples.

Sample code	Sample description	Food simulant^b^
CF 1^a^	Paper/aluminum/PE composite film for fatty products ffffffoofproducts	95% EtOH
CF 2^a^	Plastic multilayer composite film for fatty products	95% EtOH
CF 5^a^	Plastic multilayer composite film for fatty products	95% EtOH
PS 1^a^	Tray for fatty products	95% EtOH
PS 2^a^	Foil for milk products	50% EtOH
PS 3^a^	Foil for fatty products	95% EtOH
PP 1	Plastic box for microwave use	95% EtOH
PP 2	Foil for fatty products	95% EtOH
PP 7^a^	Granulate for water pipes	ultrapure water
PE 1	Granulate for water pipes	ultrapure water
PE 2^a^	Granulate for food packaging	50% EtOH
PE 3	Granulate for food packaging	95% EtOH
PET 1	Granulate for food packaging	95% EtOH
PET 2	Granulate for food packaging	95% EtOH
PET 3	0.5 liter bottle, 100% virgin material	20% EtOH
FC 1	Food carton for milk products	50% EtOH
FC 2	Food carton for milk products	50% EtOH
FC 3	Food carton for milk products	50% EtOH

a Sample was previously tested by Kirchnawy et al. [1] using the YES.

b Migration was done at 60°C for 10 days.

### Solid phase extraction

After 10 days of migration, solid phase extraction by Waters Oasis HLB columns (5 cc/200 mg) was used to extract hormone active components from the food simulants. As described in detail by Kirchnawy et al. [1] sample migrates diluted to 10% ethanol and adjusted to pH 3 were drawn through the columns and eluted into 100 µl DMSO. Elutes were evaporated under a nitrogen stream to a final volume of 150–200 µl and stored at 4°C prior to analysis by YES, YAS and CALUX bioassays. Extraction recoveries and reproducibility of the solid phase extraction were previously determined by Kirchnawy et al. [1].

### Recombinant yeast screens

The recombinant yeast strains were kindly provided by M. Wagner and J. Oehlmann from the Johann Wolfgang Goethe University Frankfurt with the permission of E.J. Routledge and J.P. Sumpter from Brunel University.

### Yeast estrogen screen

The yeast strain contains the stably integrated human estrogen receptor (hERα) gene and an expression plasmid containing the reporter gene *lacZ* under the control of estrogen responsive elements (ERE). The activation of the hERα by the binding of an estrogen active substance leads to the expression of β-galactosidase, which converts the substrate chlorophenol red-β-D-galactopyranoside (CPRG) into chlorophenol red [30].

The analysis of food contact materials was conducted as previously described by Kirchnawy et al. [1]. Briefly, yeast cells were incubated with fresh media supplemented with 1% of the respective DMSO sample extracts on a 96-well microtiter plate for 24 h. After measuring the optical density at 550 nm to ensure that sample extracts had no toxic or growth inhibiting effects on yeast cells, 100 µl *lacZ* master-mix was added to each well and the optical density was determined at 550 nm after one hour at 30 min intervals. The optical density of a serial dilution of 17β-estradiol (100 pmol/l – 10 pmol/l in the assay medium) was used as a linear calibration curve to determine the estrogen activity of each sample. The regression was calculated by MS Excel, using the formula for a linear regression curve (y = kx + d). Additionally each sample was spiked with 80 pmol/l 17β-estradiol standard and analyzed via YES, in order to detect antiestrogenic effects or potential matrix inhibition by the DMSO extracts. Good reproducibility of the YES with relatively low relative standard deviation was previously determined by Kirchnawy et al. [1]

### Yeast androgen screen

The yeast strain contains the stably integrated human androgen receptor (hAR) gene and an expression plasmid containing the reporter gene *lacZ* under the control of androgen responsive elements (ARE). The activation of the hAR by the binding of an androgen active substance leads to the expression of β-galactosidase, which converts the substrate chlorophenol red-β-D-galactopyranoside (CPRG) into chlorophenol red [31].

The analysis procedure of DMSO sample extracts in the YAS was similar to the YES with some hAR dependent variations. Instead of 17β-estradiol, 5α-dihydrotestosterone (DHT) standard (2.7 nm – 1.2 nm in the assay medium) was used as a linear calibration curve to determine the androgen activity of each sample. To test for antagonism, each sample was spiked with a non-saturating concentration of DHT (2.4 nmol/l) and analyzed via YAS, in order to detect antiandrogenic effects or potential matrix inhibition by the DMSO extracts.

To determine the reproducibility of the YAS, solvent blanks were spiked with two different concentrations of DHT (1.8 nmol/l or 2.4 nmol/l) and analyzed via bioassay. In addition, to test for a possible influence of sample matrix, the DMSO extracts of three different migrate samples (PE, PP and PET), testing negative for androgen and antiandrogen activity, were spiked with 2.4 nmol/l DHT and analyzed via YAS. Each spiked sample was analyzed five times on different days by two different operators. Standard deviations were calculated according to following equation, where N is the total number of replicates, x_i_ is the result of each individual replicate and 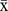 is the arithmetic mean of all replicates.
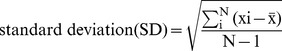



To calculate the relative standard deviation the standard deviation was divided by the arithmetic mean of all replicates (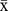).
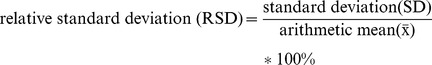



### CALUX bioassays

The genetically modified human osteoblastic osteosarcoma cell lines U2-OS were provided by BioDetection Systems (Netherlands).

### ERα CALUX

The ERα CALUX is a human U2-OS cell line stably co-transfected with an expression construct for the human estrogen receptor alpha (hERα). Activation of the hERα by the binding of an estrogen active substance leads to the transcription of luciferase, which emits light when luciferin is added as a substrate [32].

Assay procedure was conducted as previously described by Sonneveld et al. [32] with modifications. U2-OS cells were plated at a concentration of 10 000 cells/well with 100 µl phenol red-free Dulbecco's modified eagle medium (DMEM) (supplemented with dextran-coated charcoal-stripped FCS) in a 96-well microtiter plate [33]. After 24 h incubation at 37°C in a humidified atmosphere with 5% CO_2_, DMEM medium was refreshed and cells were incubated for another 24 h with 0.5% of the respective DMSO sample extract. After examining cell growth under a microscope to ensure that sample extracts had no cytotoxic or growth inhibiting effects, 50 µl Steady Glo Luciferase-Mix (Promega) was added to each well and incubated for 15 min for cell lysis. This was followed by measuring the luciferase activity of each well using a multilabel reader (Victor^3^, Perkin Elmer). The luciferase activity of a serial dilution of 17β-estradiol (10 pmol/l – 1 pmol/l in the assay medium) was used as a linear calibration curve to determine the estrogen activity of each sample. To test for antagonism, each sample was spiked with a non-saturating concentration of 17β-estradiol (approximately the EC_50_ value of 17β-estradiol, 8 pmol/l) and analyzed via ERα CALUX.

To determine the reproducibility of the ERα CALUX, three different DMSO extracts of sample migrates testing negative for estrogen and antiestrogen activity, were spiked with 8 pmol/l 17β-estradiol and analyzed via bioassay. Additional, the estrogen activity of bisphenol A at two different concentrations (0.2 µmol/l and 0.4 µmol/l) was determined. For each spiked sample and each bisphenol A concentration a three-fold determination per microtiter plate was carried out and five independent microtiter plates were analyzed by two different operators on five different days. The relative standard deviation of the determined estrogen activity was calculated for each spiked sample and for each bisphenol A concentration as described for the yeast androgen screen.

### AR CALUX

The AR CALUX is a human U2-OS osteosarcoma cell line stably co-transfected with an expression construct for the human androgen receptor (hAR). Activation of the hAR by the binding of an androgen active substance leads to the transcription of luciferase, which emits light when luciferin is added as a substrate [26].

The AR CALUX test procedure was conducted as described for ERα CALUX, except DHT (400 pmol/l - 30 pmol/l in the assay medium) was used as a reference substance, instead of 17β-estradiol, to determine the androgen activity of each sample. To test for antagonism, each sample was spiked with a non-saturating concentration of DHT (approximately the EC_50_ value of DHT, 400 pmol/l) and analyzed via AR CALUX.

To determine the reproducibility of the AR CALUX, solvent blanks were spiked with two different concentrations of DHT (200 and 400 pmol/l) and analyzed for androgen activity in the AR CALUX. In addition, to test the influence of the sample matrix the DMSO extracts of three different migrate samples (PE, PP and PET) testing negative for androgen and antiandrogen activity, were spiked with 400 pmol/l DHT and analyzed via bioassay. Each spiked sample was analyzed five times on different days by two different operators and the relative standard deviation of the results was calculated to determine the reproducibility of the method as described for the yeast androgen screen.

### GC

Gas chromatography analysis was carried out using the SBSE technique (Stir Bar Sorptive Extraction using the PDMS GERSTEL twister) in combination with thermodesorption and GC-MS. Five milliliter of each sample were extracted on a magnetic stirring plate at 2000 rpm for 1 h. The twister was removed, dried with a lint-free tissue paper and placed in a thermal desorption tube. The analyses were performed using a 6890N GC equipped with a 5975C (inert XL Triple Axis) mass selective detector (Agilent Technologies, Waldbronn, Germany), a Thermal Desorption Unit (TDU), Cooled Injection System (CIS4) and Multi-Purpose Sampler (MPS, Gerstel, Mühlheim an der Ruhr, Germany). Identification and quantification of 29 target compounds was made in selected ion monitoring (SIM) mode. The identification of unknown compounds was achieved in SCAN mode using the NIST02 library (US National Institute of Standards and Technology) and comparison to known mass spectra. Two independent migrates were each analyzed in duplicate. In order to avoid contaminations from previous tested samples twisters were conditioned at 300°C for ten minutes.

The GC-MS screening method was validated for 29 reference substances by examining the following points: linearity, precision, accuracy, limit of detection (LOD) and limit of quantification (LOQ). To test for linearity, calibration curves for each substance were constructed by plotting the peak area of the compounds versus the expected concentrations. The linear regression of each calibration curve was determined for concentrations between 1 µg/l and 50 µg/l. LOD and LOQ for each reference substance were determined according to DIN 32 645. Precision was determined using the repeatability between five replicates of a 10% ethanol sample matrix spiked with a concentration of 10 µg/l of each substance and reported as a coefficient of variation (CV). Accuracy was calculated as the percentage of recovery from three replicates at the same concentration of 10 µg/l.

### HPLC

Sample migrates were analyzed via HPLC (high performance liquid chromatography, reversed-phase chromatography) and quantified via mass spectrometry (QTrap 5500, AB Sciex) in order to screen for bisphenol A, whereby a limit of detection of less than 5 µg/l was achieved. As previously described by Kirchnawy et al. [1] an aliquot of each sample migrate was injected onto a C-18 column (ACE C18-AR (150×3.0 mm), 3 µm) and separation was achieved by increasing the initial ratio (90∶10 (A: B)) of mobile phase solvents ultrapure water (A) and ACN (B) up to an A:B ratio of 0∶100. The separated substances were compared to the multiple reaction monitoring (MRM) transitions of a generated library for qualification purposes. Quantification of bisphenol A was performed via calibration curve of a standard.

## Results

### YAS reproducibility

The relative standard deviation for an analysis of androgen activity was 15% for solvent blanks spiked with 1.8 nmol/l DHT and 12% for solvent blanks spiked with 2.4 nmol/l DHT. The YAS also showed good reproducibility when testing plastic samples with a relative standard deviation of 16% for a PP sample extract spiked with 2.4 nmol/l DHT, 13% for a spiked PET sample and 12% for a spiked PE sample.

### ERα CALUX reproducibility

The relative standard deviation was 13% for the analysis of estrogen activity of 0.2 µmol/l bisphenol A and 6% for the analysis of 0.4 µmol/l bisphenol A. The bioassay also showed good reproducibility when testing plastic samples with a relative standard deviation of 8% for a PP sample extract spiked with 8 pmol/l estradiol, 9% for a spiked PET sample and 11% for a spiked PE sample.

### AR CALUX reproducibility

The relative standard deviation was 13% for a solvent blank spiked with 100 pmol/l DHT and 14% for a solvent blank spiked with 400 pmol/l DHT. The relative standard deviation was between 14% for the PP sample extract spiked with 300 pmol/l DHT and 19% for the spiked PE sample. With a relative standard deviation of 17%, the PET sample was in between.

### GC validation

Linearity (r>0.99) obtained for each reference substance calibration curve was found to be sufficient for method application. LOD was determined to be equal or less than 3 µg/l, whereas LOQ was below 10 µg/l for each compound (LOD and LOQ were ten times higher for 95% ethanol migrates and five times higher for 50% ethanol migrates due to a 1∶10 or 1∶5 dilution of each migrate prior to GC analysis). For all substances tested in the bioassays (shown in Table 2 and Table 3) the observed hormone activities are much lower than the activities of the reference substances 17β-estradiol and DHT. Following, the determined LOD and LOQ for GC analysis are sensitive enough to cover the substances at a concentration level which shows an activity in the bioassays. The coefficient of variation for the determination of precision did not exceed 15%. Recovery for the determination of accuracy was between 90 and 115% with the exception of diethylphthalate which showed a recovery of approximately 150%.

**Table 2:** Estrogen and antiestrogen activities of identified substances in the YES and ER CALUX.

Substance	CAS#	YES		ER CALUX	
		Estrogen activity EC_50_ [M]	Antiestrogen activity EC_50_ [M]	Estrogen activity EC_50_ [M]	Antiestrogen activity EC_50_ [M]
*diethylhexyladipate* (DEHA)	**103-23-1**	**>1×10^−3^**	**>1×10^−3^**	**>5×10^−4^**	**>5×10^−4^**
*2,6-bis(1,1-dimethylethyl)-4-methylphenol* (BHT)	**128-37-0**	**>1×10^−3^**	**>1×10^−3^**	**>5×10^−5^** a	**>5×10^−5^** a
*1,3-diphenylpropane*	**1081-75-0**	**5×10^−4^**	**>1×10^−3^**	**4×10^−5^**	**>5×10^−4^**
*2,4-di-cumylphenol*	**2772-45-4**	**6×10^−4^**	**>3×10^−4^** b	**5×10^−6^**	**>2×10^−5^** a
*2,4-di-tert-butylphenol*	**96-76-4**	**>1×10^−5^** b	**>1×10^−5^** b	**>2×10^−5^** a	**>2×10^−5^** a
*17β-estradiol*	**50-28-2**	6 *10^−11^	**-**	**1×10^−11^**	**-**
*4-ortho hydroxytamoxifen*	**68392-35-8**	**-**	**4×10^−6^**	**-**	**2×10^−10^**

CAS#… Chemical Abstracts Service Number.

a … inhibition of human U2-OS osteosarcoma cell growth at higher concentrations.

b … inhibition of yeast growth at higher concentrations.

**Table 3:** Androgen and antiandrogen activities of identified substances in the YAS and AR CALUX.

Substance	CAS#	YAS		AR CALUX	
		Androgen activity EC_50_ [M]	Antiandrogen activity EC_50_ [M]	Androgen activity EC_50_ [M]	Antiandrogen activity EC_50_ [M]
*diethylhexyladipate* (DEHA)	**103-23-1**	**>1×10^−3^**	**>1×10^−3^**	**>5×10^−4^**	**>5×10^−4^**
*2,6-bis(1,1-dimethylethyl)-4-methylphenol* (BHT)	**128-37-0**	**>1×10^−3^**	**>1×10^−3^**	**>5×10^−5^** a	**>5×10^−5^** a
*1,3-diphenylpropane*	**1081-75-0**	**>1×10^−3^**	**3×10^−5^**	**>5×10^−4^**	**3×10^−5^**
*2,4-di-cumylphenol*	**2772-45-4**	**>1×10^−3^**	**2×10^−5^**	**>5×10^−6^** a	**>5×10^−6^** a
*2,4-di-tert-butylphenol*	**96-76-4**	**>1×10^−5^** b	**>1×10^−5^** b	**>2×10^−5^** a	**>2×10^−5^** a
5α-dihydrotestosterone	**521-18-6**	**2×10^−9^**	**-**	**4×10^−10^**	**-**
*flutamide*	**13311-84-7**	**-**	**4×10^−6^**	**-**	**1×10^−6^**

CAS#… Chemical Abstracts Service Number.

a … inhibition of human U2-OS osteosarcoma cell growth at higher concentrations.

b … inhibition of yeast growth at higher concentrations.

### Samples tested with YES and ERα CALUX

DMSO extracts were screened in triplicate analysis for estrogen and antiestrogen activity using the YES and ERα CALUX bioassay. Samples which showed growth inhibition, toxic or antagonistic effects were diluted for analysis resulting in a higher limit of detection. By comparison with a 17β-estradiol standard curve the estrogen activity of each sample was converted into EEQ (presented in Table 4). Since the amount of food simulant varied between each plastic sample, and 95% and 50% ethanol migrates were diluted before they were extracted with solid phase extraction, different LODs were determined for each sample.

**Table 4:** Estrogen and antiestrogen activity of migrates from plastic samples.

Sample code	YES			ERα CALUX		
	EEQ [ng/l]	LOD [ng EEQ/l]	Antiestrogen activity	EEQ [ng/l]	LOD [ng EEQ/l]	Antiestrogen activity
CF 1	59.6±29.3	1	−	21.3±7.1	0.2	−
CF 2	<LOD	1	−	< LOD	0.2	−
CF 5	11.1±3.2	1	−	11.3±2.6	0.2	−
PS 1	2.1±0.6	1	−	2.6±0.4	0.2	−
PS 2	< LOD^a^	4	++	3.3±0.9	0,1	−
PS 3	< LOD^b^	8	−	3.1±0.7	0,2	−
PP 1	< LOD	1	−	< LOD	0.2	−
PP 2	< LOD	1	−	< LOD	0,2	−
PP 7	< LOD^a^	1	+	< LOD	0.02	−
PE 1	< LOD^a^	0,8	+	< LOD	0.02	−
PE 2	0.7±0.2	0.5	−	0.3±0.1	0.1	−
PE 3	< LOD	1	−	< LOD	0.2	−
PET 1	< LOD	1	−	< LOD	0.2	−
PET 2	< LOD	1	−	< LOD	0.2	−
PET 3	< LOD	0,2	−	< LOD	0.04	−
FC 1	< LOD^a^	10	+++	< LOD	0.1	−
FC 2	< LOD	0.5	−	< LOD	0.1	−
FC 3	< LOD^a^	8	++	< LOD	0.1	−

a … inhibition of response to 17β-estradiol in undiluted samples.

b … inhibition of yeast growth in undiluted samples.

−… no antiestrogen activity was detected.

+… antiestrogen activity in the range of the activity of 0.01 to 0.1 mg/l 4-OHT.

++… antiestrogen activity in the range of the activity of 0.1 to 1 mg/l 4-OHT.

+++… antiestrogen activity in the range of the activity of 1 to 10 mg/l 4-OHT.

Estrogenic activity was detected in four samples out of 18 tested using the YES and ERα CALUX. In the ERα CALUX two additional samples showed estrogen activity which was below the LOD of the YES, due to the dilution of the DMSO extracts. The highest estrogen activities of 59±29 ng EEQ/l and 21±7 ng/l measured in the YES and ERα CALUX respectively, were detected for sample CF1. In general, estrogen activities of samples testing positive were in the same order of magnitude for both bioassays as shown in Table 4.

**Figure 1:**
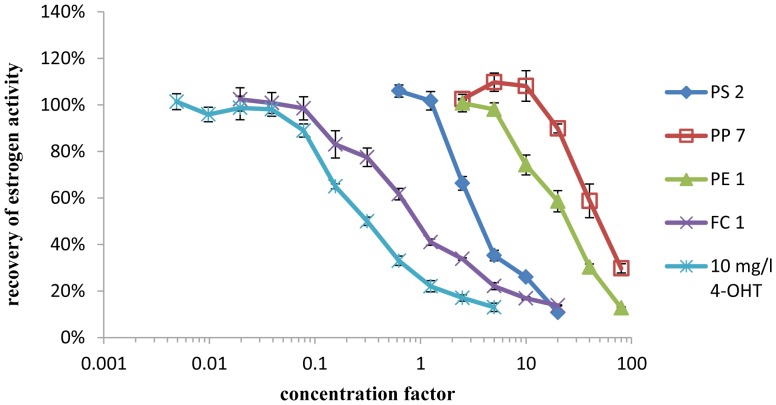
Antiestrogenic activity of samples spiked with 80/l 17β-estradiol standard as measured by the YES.

**Figure 2:**
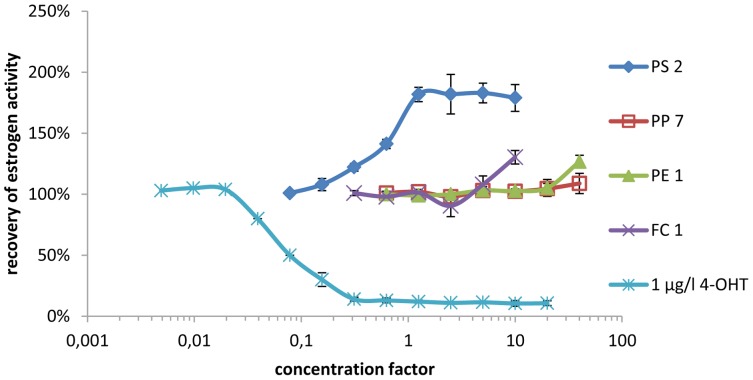
Antiestrogenic activity of samples spiked with 8/l 17β-estradiol standard as measured by ERα CALUX.

In the yeast estrogen screen antagonistic effects could be observed in five out of 18 samples tested. The antiestrogen activity of these samples was compared to the antiestrogen activity of 4-OHT, the active metabolite of the pharmaceutical antiestrogen tamoxifen. By comparison of a dilution series of each sample to the EC_50_ of 4-OHT, the antiestrogen activity was categorized using the symbols “+” for sample migrates with an antiestrogen activity in the range of the activity of 0.01 to 0.1 mg/l 4-OHT, “++” for migrates with an activity in the range of 0.1–1 mg 4-OHT equivalents/l and “+++” for samples with an activity in the range of 1–10 mg 4-OHT equivalents/l (shown in Table 4). The antagonistic effects of all samples were reversed when the estrogen concentration was increased to 5 nmol/l 17β-estradiol (which is approximately 100 times the EC_50_ value), indicating that the observed antagonistic effects are specific to the estrogen receptor (data not shown). However, the antiestrogenic activity detected in the YES (shown in Figure 1) could not be verified using ERα CALUX (shown in Figure 2). None of the solvent blanks tested showed estrogenic activity in the YES or ERα CALUX.

### Samples tested with YAS and AR CALUX

Androgen and antiandrogen activity of DMSO sample extracts were determined using YAS and AR CALUX bioassay. For quantification, the androgen activity each sample was compared to a DHT standard curve. Samples with antagonistic or inhibiting effects were diluted prior to analysis for androgen activity resulting in a higher LOD.

**Figure 3:**
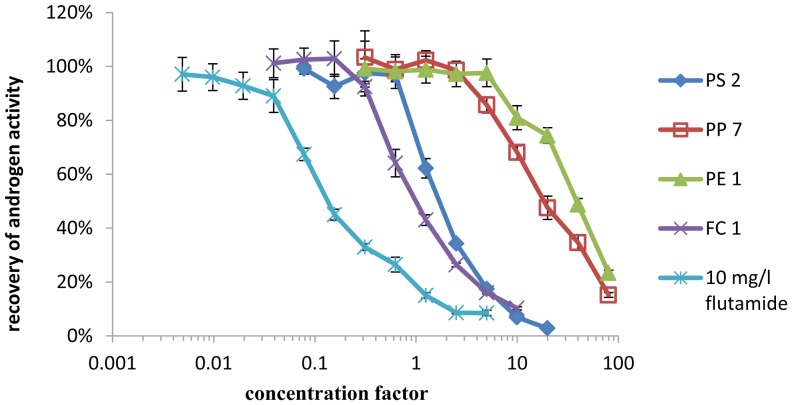
Antiandrogenic activity of samples spiked with 2.4/l DHT standard as measured by the YAS.

**Figure 4:**
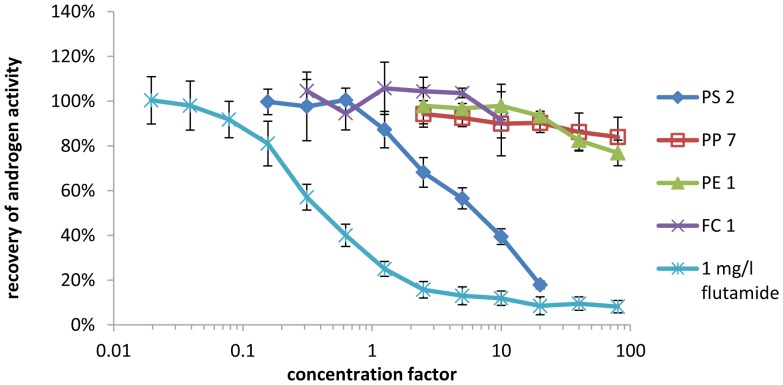
Antiandrogenic activity of samples spiked with 400/l DHT standard as measured by AR CALUX.

None of the samples showed androgen activity in either YAS or AR CALUX. One sample tested positive for antiandrogen activity in both bioassays as shown in Figure 3 and Figure 4. For nine additional samples, antiandrogen activity was determined in the YAS, but was not confirmed in AR CALUX, as shown in Table 5. Flutamide was used as a reference compound for the semi-quantification of antiandrogen activity. The antiandrogen activity of these samples was compared to the activity of the pharmaceutical androgen antagonist flutamide. By comparison of the EC_50_ of a dilution series of each sample to the EC_50_ of flutamide, the antiandrogen activity was categorized using the symbols “+” for sample migrates with an antiandrogen activity in the range of the activity of 0.01 to 0.1 mg/l flutamide, “++” for migrates with an activity in the range of 0.1–1 mg flutamide equivalents/l and “+++” for samples with an activity in the range of 1–10 mg flutamide equivalents/l (shown in Table 5). The antagonistic effects of all samples were reversed when the androgen concentration was increased to 240 nmol/l DHT (which is approximately 100 times the EC_50_ value), indicating that the observed antagonistic effects are specific to the androgen receptor (data not shown). None of the solvent blanks tested showed androgen activity in the YAS or AR CALUX.

**Table 5:** Androgen and antiandrogen activity of migrates from plastic samples.

Sample code	YAS			AR CALUX		
	AEQ [ng/l]	LOD [ng AEQ/l]	Antiandrogen activity	AEQ [ng/l]	LOD [ng AEQ/l]	Antiandrogen activity
CF 1	< LOD^a^	200	+	< LOD	4	−
CF 2	< LOD	50	−	< LOD	4	−
CF 5	< LOD	200	+	< LOD	4	−
PS 1	< LOD^a^	200	+	< LOD	4	−
PS 2	< LOD^a^	400	++	< LOD^a^	20	++
PS 3	< LOD^b^	50	−	< LOD	4	−
PP 1	< LOD	50	−	< LOD	4	−
PP 2	< LOD^a^	200	+	< LOD	4	−
PP 7	< LOD^a^	50	+	< LOD	0.4	−
PE 1	< LOD^a^	20	+	< LOD	0.4	−
PE 2	< LOD	50	+	< LOD	2	−
PE 3	< LOD	50	−	< LOD	4	−
PET 1	< LOD	50	−	< LOD	4	−
PET 2	< LOD	50	−	< LOD	4	−
PET 3	< LOD	10	−	< LOD	0.8	−
FC 1	< LOD^a^	800	+++	< LOD	2	−
FC 2	< LOD	25	−	< LOD	2	−
FC 3	< LOD^a^	400	++	< LOD	2	−

a … inhibition of response to DHT in undiluted samples.

b … inhibition of yeast growth in undiluted samples.

−… no antiandrogen activity was detected.

+… antiandrogen activity in the range of the activity of 0.01 to 0,1 mg/l flutamide.

++… antiandrogen activity in the range of the activity of 0.1 to 1 mg/l flutamide.

+++… antiandrogen activity in the range of the activity of 1 to 10 mg/l flutamide.

### Samples analyzed with GC-MS

In order to identify hormone active substances, samples testing positive in the yeast or CALUX assays were analyzed by GC-MS. Identification and quantification of 29 target compounds were made in SIM mode, whereas identification of unknown peaks was achieved in SCAN mode by a database comparison of the mass spectra. Table 6 shows all substances that were detected in sample migrates by SIM mode, and additional substances that were identified in SCAN mode showing a mass spectra similarity of more than 90%. With diethylhexyladipate in sample CF 1 and 2,4-di-tert-butylphenol in sample PP 2 only two substances could be detected in SIM mode. In all other tested samples target compounds were below the LOD listed in Table 7. In addition to the detected target compounds some substances with suspected hormone activity were identified in SCAN mode using the NIST02 library. In all samples additional peaks were detected that could not be identified with sufficient similarity to known mass spectra. None of the substances listed in Table 7 was found in any of the solvent blanks.

**Table 6:** Identification of substances in migrates of samples which were hormone active in bioassay analysis.

Sample	Identified compound	CAS#	Retention time [min]	Concentration in migrate [mg/l]
CF 1	***diethylhexyladipate*** ** (DEHA)**	**103-23-1**	**24.2**	**1.2±0.4**
CF 5	***2,6-bis(1,1-dimethylethyl)-4-methylphenol*** ** (BHT)** b	**128-37-0**	**15.2**	**-**
PS 1	***1,3-diphenylpropane*** a	**1081-75-0**	**17.2**	**-**
	***1,2-diphenylcyclobutane*** b	**20071-09-4**	**18.4**	**-**
	***1,2-dihydro-3-phenylnaphtalene*** b	**20669-52-7**	**18.8**	**-**
PS 2	***1,3-diphenylpropane*** a	**1081-75-0**	**17.2**	**-**
	***1,2-diphenylcyclobutane*** b	**20071-09-4**	**18.4**	**-**
	***1-phenyl-1,2,3,4-tetrahydronaphthalene*** b	**3018-20-0**	**18.6**	**-**
PS 3	***1,3-diphenylpropane*** a	**1081-75-0**	**17.2**	**-**
	***1,2-diphenylcyclobutane*** b	**20071-09-4**	**18.4**	**-**
PE 2	***2,4-di-cumylphenol*** b	**2772-45-4**	**24.9**	**-**
PP 2	***2,4-di-tert-butylphenol***	**96-76-4**	**15.4**	**0.11±0.03**

a … identification of the substance verified by comparison to a standard.

b … identification of the substances by database comparison of mass spectra, not verified by comparison to a standard.

LOD…Limit of detection.

LOQ…Limit of quantification.

CAS#… Chemical Abstracts Service Number.

**Table 7:** Validation parameters of GC-MS (SIM) analysis of 29 reference substances.

Reference substance	CAS#	Correlation coefficient (r)	LOD [µg/l]	LOQ [µg/l]	CV [%]	Recovery [%]
dicyclohexylphthalate	84-61-7	0.9980	1.4	5.1	6.9	96.4±6.3
diethylphthalate	84-66-2	0.9983	1.5	5.5	12.4	154±20.3
2,4-di-tert-butylphenol	96-76-4	0.9937	0.6	2.1	11.1	95.1±2.6
dibutylphthalate (DBP)	84-74-2	0.9934	2.6	9.1	12.7	89.2±4.7
di-n-hexylphthalate (DnHP)	84-75-3	0.9973	1.7	5.9	7.1	94.1±6.5
butyl benzyl phthalate (BBP)	85-68-7	0.9987	1.1	4.0	5.5	93.6±4.8
bis (2-ethylhexyl) phtalate (DEHP)	117-81-7	0.9976	1.6	5.5	7.6	95.3±9.3
4-chloro-3-methyl-phenol	59-50-7	0.9990	1.0	3.6	4.8	108.2±6.5
4-methylbenzophenone	134-84-9	0.9964	1.9	6.7	9.3	98.4±2.5
4,4′-thiobis(6-tert-butyl-3-methyl-phenol)	96-69-5	0.9972	1.7	5.8	8.2	104.1±7.1
2-phenylphenol	90-43-7	0.9986	1.2	4.2	5.9	111.4±2.6
4-phenylphenol	92-69-3	0.9966	1.8	6.6	9.0	106.3±8.4
4-nonylphenol (NP)	104-40-5	0.9968	2.1	7.4	10.0	95.2±5.4
2,2′-methylenebis(4-ethyl-6-tert-butylphenol)	88-24-4	0.9973	1.7	6.0	8.4	94.5±3.0
2,2′-methylene bis(4-methyl-6-tert-butylphenol)	119-47-1	0.9960	2.1	7.3	10.2	93.9±6.2
p-cumylphenol	599-64-4	0.9956	2.4	8.4	11.8	96.3±9.9
triclosan	3380-34-5	0.9970	1.7	6.2	8.5	98±4.9
BHA	25013-16-5	0.9979	1.6	5.9	8.1	108.4±2.5
ethyl-4-hydroxy-benzoate (ethylparaben)	120-47-8	0.9864	4.3	8.0	10.3	107.2±5.9
n-propyl-p-hydroxybenzoate (propylparaben)	94-13-3	0.9943	2.4	8.6	11.8	106±7.1
methyl p-hydroxybenzoate (methylparaben)	99-76-3	0.9965	1.9	6.7	9.2	112.1±10.8
diethylhexyl adipate	103-23-1	0.9966	1.9	6.5	9.0	93.3±4.9
diphenyl-p-phenylenediamine	74-31-7	0.9911	3.0	9.7	14.7	98.2±10.1
oleamide	301-02-0	0.9955	2.1	7.6	10.4	108.1±5.8
1,4-dichlorobenzene	106-46-7	0.9978	1.5	5.3	7.4	103.1±3.8
benzophenone	119-61-9	0.9970	1.7	6.2	8.6	106.3±9.9
2,20-dihydroxy-4-methoxybenzophenone	131-53-3	0.9944	2.4	8.2	11.6	114.8±7.1
2,4-dihydroxybenzophenone	131-56-6	0.9937	2.8	9.9	13.8	90.6±5.0
oxybenzone	131-57-7	0.9979	1.4	5.1	10.0	104.2±0.8

In samples PP 7, FC 1 and FC 3 no substances were detected in SIM mode and no additional substances could be identified in SCAN mode.

### Samples analyzed with HPLC-MS

All samples that showed an activity in the bioassays were screened for bisphenol A by HPLC-MS. Bisphenol A was not detected in any of the samples with a LOD of 5 µg/l.

## Discussion

The anti(estrogen) and anti(androgen) activity of different food packaging was characterized using a combination of validated bioassays (YES, YAS, ERα CALUX and AR CALUX) and chemical trace analysis via HPLC-MS and GC-MS. For each hormone axis two independent bioassays were used to characterize the hormone activity of the samples. Results of yeast based screening methods (YES and YAS) were compared with results obtained with human cell-based bioassays (ERα CALUX and AR CALUX).

### Hormone activity of samples detected with bioassays

By screening for estrogen and androgen agonists we could determine a high level of agreement between bioassays. Four (CF 1, CF 5, PS 1 and PE 2) out of 18 samples showed estrogen activity in a similar range in both YES and ERα CALUX (shown in Table 4). With 59 ng EEQ/l (YES) and 21 ng EEQ/l (ERα CALUX), the highest estrogen activity of all food contact material migrates was detected in CF 1. CF 5 followed with an estrogen activity of 11.1 ng EEQ/l (YES) and 11.3 ng EEQ/l (ERα CALUX). PS 1 and PE 2 showed low estrogen activity in both bioassays. Two additional samples (PS2 and PS3) that tested negative in the YES, showed estrogen activity in the ERα CALUX bioassay. However, activities detected with ERα CALUX were below the limits of detection in the YES, as these two samples had to be diluted prior to analysis due to antagonistic or toxic effects occurring in the YES. Androgen agonists could not be detected in any of the tested samples, neither with YAS nor with AR CALUX.

When testing for antagonists, significant differences between yeast and human cell-based bioassays were observed. In the YES, five samples showed strong antagonistic effects equal to the antagonistic effect of more than 1 mg/l (387 mmol/l) of the pharmaceutical estrogen antagonist 4-OHT which is already a clinical relevant dose [34]. As no reduction of cell growth was observed, the suppression of response to 17β-estradiol cannot be caused by toxic or growth inhibiting effects of the samples. In additional experiments when the DMSO extracts were coincubated with excess 17β-estradiol (100 times the EC_50_ value) the antagonistic effects were reversed. Therefore many unspecific effects (for example suppression of the β-galactosidase reaction or unspecific suppression of reporter gene expression) can be excluded as an explanation for the suppression of hormone activity observed in the bioassay.

However, an analysis of the same samples with the human cell-based ERα CALUX assay did not show any antiestrogenic effects, not even when the concentration of the DMSO extract in the ERα CALUX was increased to 1% to ensure direct comparability with the yeast estrogen screen. These results clearly demonstrate that the observed suppression of hormone activity is specific to the yeast cell-based YES.

All samples that showed antiestrogen activity in the YES also inhibited the response to DHT in the YAS, which indicates that the observed effect is not specific to the estrogen receptor. Two additional samples that were estrogen active in the YES showed antiandrogen activity in the YAS. This matches with results of Sohoni and Sumpter [31], who showed that many substances that are estrogen active in the YES show antiandrogen activity in the YAS.

However, antagonistic effects could only be verified for one sample (PS 2) by analysis via AR CALUX, whereas the other six samples tested negative for antiandrogen activity. The antiandrogen activity of sample PS 2 was in the range of the activity of 0.1–1 mg/l (27.6–276 mmol/l) of the pharmaceutical antiandrogen flutamide.

Our results show that migrates of polymer samples are often antagonistic in both YES and YAS which matches with the results of Wagner and Oehlmann [21] who detected antagonistic effects in 50% of all analyzed plastic samples in YAS, and in 44% of all tested packaging samples in YES. However, a comparison to the results with the CALUX assays shows that most of the antagonist effects are specific for the recombinant yeast screens YES and YAS. The validation of the bioassays confirms that all four *in vitro* assays are suitable for a screening of food contact material migrates for estrogen and androgen agonists. LODs were significantly lower for CALUX assays, particularly because many samples had to be diluted in the YES and YAS due to antagonistic effects that seem to be specific for the yeast tests. The high occurrence of antagonistic effects in YES and YAS when testing food contact materials, shows the necessity of performing additional spiking experiments for antagonistic effects to prevent false negative results.

### GC-MS screening of migrates

Each migrate of plastic packaging that showed activity in any of the bioassays was further analyzed by GC-MS and HPLC-MS in order to identify hormone active or hormone suppressing substances.

#### Sample CF 1

**Figure 5:**
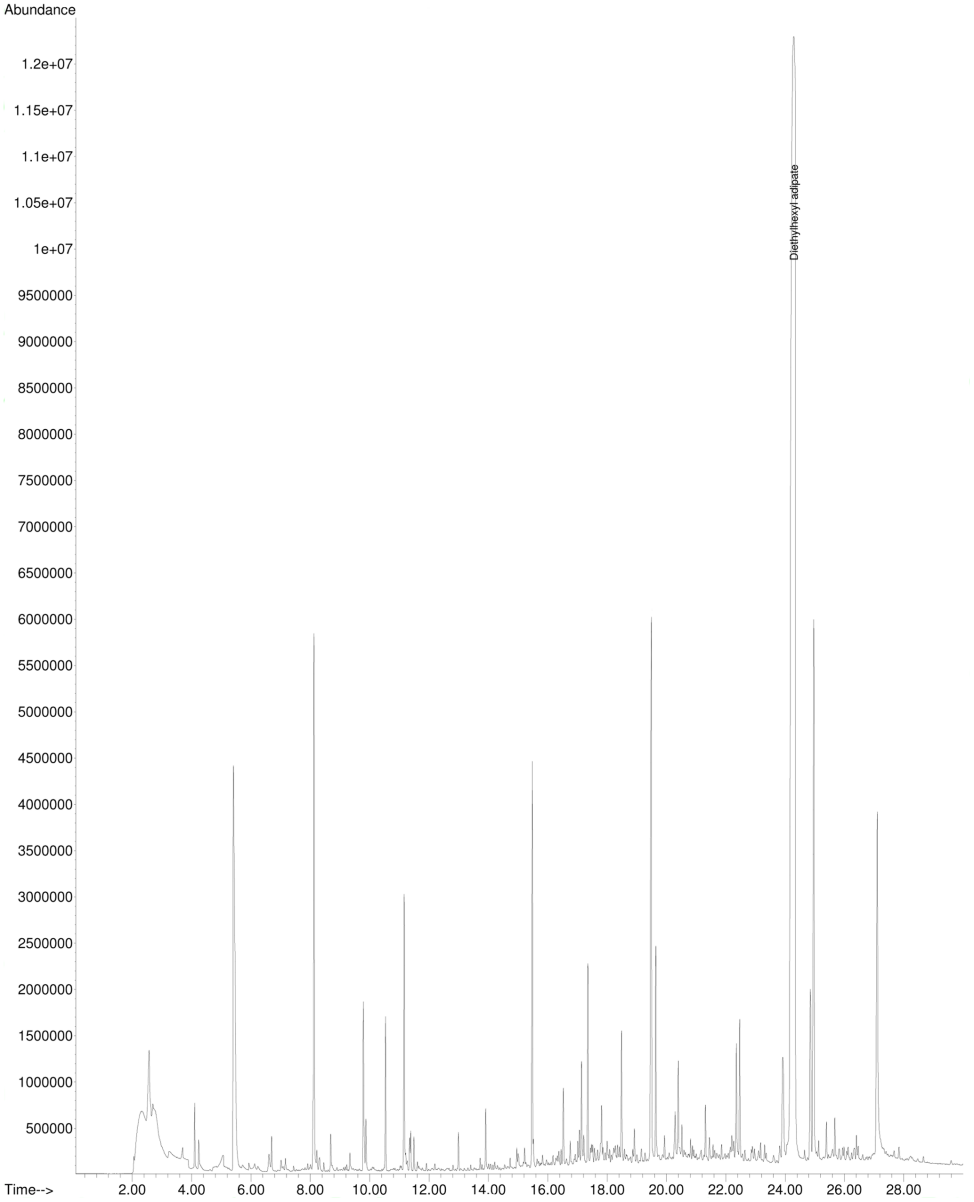
GC-MS-Analysis: Total Ion Chromatogram (TIC) of a migrate of sample CF 1.

**Figure 6:**
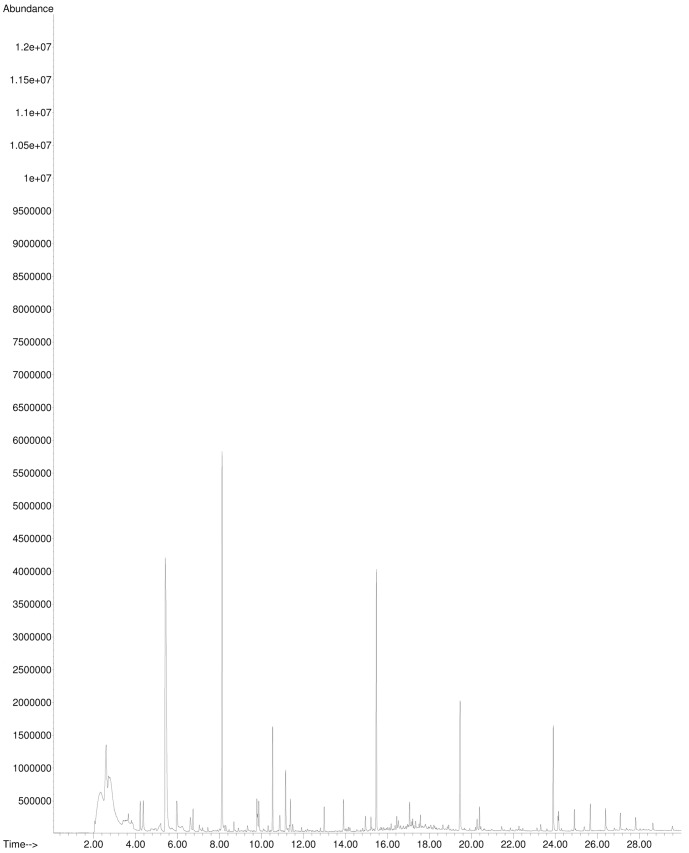
GC-MS-Analysis: Total Ion Chromatogram (TIC) of a sample blank.

The plasticizer diethylhexyladipate was the only substance that was identified in sample migrates (compared to a standard) by GC-MS. According to Wagner and Oehlmann [18] diethylhexyladipate tested positive for estrogen activity using the E-Screen. However, diethylhexyladipate did not show any activity in our bioassays, YES and ERα CALUX. Except for diethylhexyladipate no other substances that are suspected of being hormone active could be detected using the single ion mode (SIM) to screen 29 target compounds. As shown in Figure 5, many peaks were detected in SCAN mode. Most of these peaks were identified as siloxanes from the column and the twister and were also present in the blank (Figure 6). However, also some additional peaks that were solely present in the sample migrate could be detected but not identified by database comparison.

#### Sample CF 5

The antioxidant BHT was identified in sample CF 5. According to Jobling et al. [35], BHT tested positive for estrogen at a concentration of 100 µmol/l using a kidney fibroblast cell 293T and human breast cancer cells ZR-75. We could not detect any estrogen activity of BHT using YES and ERα CALUX bioassays.

#### Sample PS 1

Two different styrene dimers (1,3-diphenylpropane and 1,2-diphenylcyclobutane) that are suspected of being estrogen active were detected by GC-MS analysis. As shown in Table 2 1,3-diphenylpropane tested positive for estrogen activity in YES and ERα CALUX. 1,2-diphenylcyclobutane could not be purchased and analyzed as a standard, but was previously tested by Ohyama et al. [36] to be 4*10^5^ times less active than 17β-estradiol in a human cell-based MCF7 proliferation assay. For 1,2-dihydro-3-phenylnaphtalene, a third styrene dimer that was detected, no information is available about a possible estrogen activity.

#### Sample PS 2

The three substances 1,3-diphenylpropane, 1,2-diphenylcyclobutane and 1-phenyl-1,2,3,4-tetrahydronaphthalene were detected in sample PS 2 by chemical trace analysis. 1,3-diphenylpropane showed estrogen and antiandrogen activity as shown in Table 2 and Table 3. There is currently no data available concerning a possible hormone activity of 1-phenyl-1,2,3,4-tetrahydronaphthalene, which is a Diels-Alder dimer of styrene [37].

#### Sample PS 3

The two styrene dimers 1,3-diphenylpropane and 1,2-diphenylcyclobutane were also detected in sample PS 3.

#### Sample PE 2

2,4-di-cumylphenol, a possible degradation product of the antioxidant bis(2,4-dicumylphenyl)-pentaerythritol-diphosphite Alkanox 28, detected by GC-MS, showed estrogen activity in the ERα CALUX and the YES (shown in Table 2) and could be a source of hormone activity.

#### Sample PP 2

In sample PP 2 2,4-di-tert-butylphenol was detected at a concentration of 0.11 mg/l. 2,4-di-tert-butylphenol has been shown to be an androgen antagonist in CHO-K1 cells and in rainbow trout [38], [39]. However, in our own tests 2,4-di-tert-butylphenol was not antiandrogen active in either YAS or AR CALUX.

In samples PP 1, FC 1 and FC 3 that showed antagonistic effects in the yeast screens which could not be verified by CALUX bioassays, no potentially antiestrogen and antiandrogen active substances were detected by GC-MS and HPLC-MS. The source of the observed antagonistic effects, which seem to be yeast specific, remains unclear. As a result of the strong antagonistic activity, particularly in sample FC 1, high concentrations of hormone antagonists would be needed to explain the observed activity, even if the antagonist has an activity equal to the activity of the pharmaceutical antagonists, flutamide and 4-OHT. It seems more likely that the effects are caused by unspecific effects such as dissolved polymer interfering with yeast cells. This presumption is further supported as presently no antiestrogen active polymer compounds are known, and Sohoni and Sumpter [31] for example did not detect any antiestrogen activity by analyzing potential hormone active reference substances in the YES.

## Conclusion

The aim of the study was to further characterize preselected samples from a previous screening of food contact materials by Kirchnawy et al. [1] on their estrogen and androgen activity using four different bioassays (YES, YAS, ERα and AR CALUX) in combination with chemical trace analysis by GC-MS and HPLC-MS. A comparison of the results of the different bioassays shows that antagonistic effects occurring in the yeast cell based test systems (YES, YAS) could not be detected by human cell based bioassays (ERα and AR CALUX), although they are more sensitive to receptor antagonists as shown for the reference standards 4-ortho hydroxytamoxifen and flutamide in Table 2 and Table 3. For a screening of food contact materials for hormone activity the primary purpose is to cover potential impacts on humans whereby testing for antagonists can lead to false positive results in YES and YAS. Furthermore the high occurrence of antagonistic effects in YES and YAS when examining food contact materials, shows the necessity of performing additional spiking experiments to prevent false negative results when testing for agonists. Consequently, following our experiences, although more laborious, human cell based reporter gene assays are more suitable to analyze food contact materials for hormone activity than the yeast cell based test systems.

In general it is not clear if the observed activities in *in vitro* tests were indeed caused by the substances detected by chemical analysis. Beside substances approved for food contact use many non-intentionally added substances, which include master batch impurities, side products from complex polymerization reactions or breakdown products from antioxidants, are present in food packaging and might be a source of detected hormone activity [40]. Some non-intentionally added substances could be identified by chemical analysis, for example 1,3-diphenylpropane and 1,2-diphenylcyclobutane, common by-products of polystyrene polymerization [41]. Further research is required to constantly update and extend the list of EAS that are responsible for hormonal activity in the *in vitro* tests.

Our analyses focused on specific selected samples and do not represent a general market analysis of food contact materials. The hereby detected estrogen and antiandrogen activities were lower than expected based on previous studies on mineral water [16], [17], [42] and are according to today's state of knowledge not considered to be a threat to consumers [42]. However, no direct conclusions about potential human health effects can be drawn, as there are many aspects of endocrine action, such as metabolism, bioavailability or tissue and cellular-context dependent effects of the steroid receptors that cannot be covered by the reporter gene assays used in this study [43]–[46].
